# Unilateral Recurrent Anterior Uveitis as the Presenting Sign of Bladder Carcinoma

**DOI:** 10.4274/tjo.92259

**Published:** 2016-08-15

**Authors:** Günhal Şatırtav, Meryem Donbaloğlu, Refik Oltulu, Pembe Oltulu, Hürkan Kerimoğlu, Ahmet Özkağnıcı

**Affiliations:** 1 Necmettin Erbakan University Meram Faculty of Medicine, Department of Ophthalmology, Konya, Turkey; 2 Necmettin Erbakan University Meram Faculty of Medicine, Department of Pathology, Konya, Turkey

**Keywords:** Anterior uveitis, bladder carcinoma, paraneoplastic syndrome

## Abstract

A 79-year-old male patient was followed for unilateral uveitis with 3 attacks in 10 months, despite initial improvement with steroid therapy. The patient had visual acuity (VA) of counting fingers in right eye, hypopyon and vitritis with no chorioretinal lesions. The left eye was normal. The patient was evaluated for intraocular foreign body, intraocular lymphoma and associated systemic disease and malignancy. Computed tomography of the abdomen showed a mass in the bladder. Biopsy confirmed bladder carcinoma. After resection of the mass, intraocular inflammation improved completely and no attack was noted in the follow-up. In his last examination, two years after the operation, VA was light perception; seclusio pupilla and mature cataracts were seen on biomicroscopy. There was no sign of vitritis on ocular ultrasonography. Evidence is discussed that suggests a link and potential etiology between refractory uveitis with hypopyon and bladder carcinoma. This is the first case of unilateral recurrent uveitis with hypopyon as the initial presenting sign of bladder carcinoma.

## INTRODUCTION

The association between uveitis and concurrent malignancies has been described as pseudouveitis. Masquerade syndromes are a group of disorders that mimic ocular inflammatory disease, which are thought to be immune responses to concurrent intraocular tumors.^1^ Masquerade syndromes usually do not respond well to steroid treatment. Another possible diagnosis to be considered is the paraneoplastic syndrome, the remote effect of a systemic malignancy presenting as ocular inflammatory disease. The pathogenesis of paraneoplastic syndromes is thought to be autoimmune, and they also do not respond well to steroid treatment.^2^

To our knowledge, the presentation of bladder carcinoma with unilateral anterior uveitis with hypopyon has not been reported to date. Here we report a case in which unilateral uveitis led to the detection of bladder carcinoma.

## CASE REPORT

A 79-year-old male patient presented with loss of vision in the right eye for the previous 4 days. Ophthalmic examination revealed corrected visual acuity (VA) of counting fingers in the right eye and 20/20 in the left eye. On biomicroscopic examination, marked conjunctival hyperemia with remarkable fibrin strands and hypopyon in the anterior chamber were detected in the right eye, while the anterior segment of the left eye was normal. The fundus of the right eye could not be examined because of fibrin strands in the anterior chamber, but ocular ultrasonography showed no vitreous inflammation. Specific tests such as peripheral blood count, serum liver enzyme levels, rheumotological markers, bone marrow cytology with cellular morphology, pathergy test and chest radiography were performed to rule out any concurrent rheumatologic disease. We ruled out the presence of an intraocular foreign body with a computed tomography scan and orbital and cranial magnetic resonance imaging for possible intraocular or central nervous system lymphoma was performed. The results did not reveal any pathology. He was initially prescribed topical 0.1% dexamethasone sodium phosphate eye drops (Dexasine, Liba Lab., İstanbul, Turkey) hourly, tropicamide (Tropamid, Bilim Drug, İstanbul, Turkey) and 1% cyclopentolate HCl eye drops (Sikloplejin, Abdi İbrahim Drug Co., İstanbul, Turkey) three times daily and subconjunctival dexamethasone (Dekort, Deva Holding, İstanbul, Turkey) injections twice a day. At follow-up 5 days later, reduction of anterior chamber reaction and hypopyon was observed. The topical and subconjunctival steroid treatments were tapered. The hypopyon and anterior chamber reaction completely resolved in two weeks.

**Figure 1:**
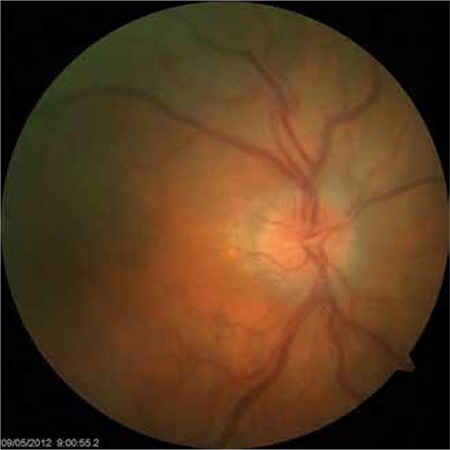
Fundus photograph of the right eye showing mild vitritis with increase in vascular tortuosity

**Figure 2:**
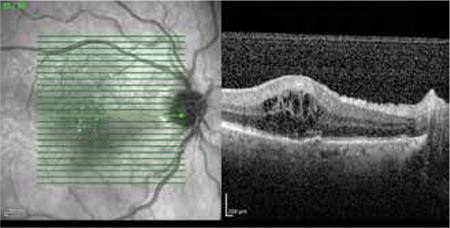
Optical coherence tomography appearance of the cystoid macular edema in the right eye

One month later in the follow-up, the patient presented with anterior chamber reaction with hypopyon once again. Best corrected VA (BCVA) was 20/200 in the right eye and 20/20 in the left eye. After one week of topical steroid treatment, VA increased to 20/63 and intraocular inflammation improved significantly. The patient continued to use topical steroids but, despite the initial improvement, two weeks after the onset of the second attack his vision deteriorated and the anterior chamber reaction increased. Fundus examination revealed moderate vitritis, cystoid macular edema (CME) and increased vascular tortuosity (Figure 1). Ocular coherence tomography (Heidelberg Spectralis, Heidelberg Engineering, Germany), confirmed CME in the right eye (Figure 2). Fundus fluorescein angiography showed leakage at the optic disc, leakage with “flower pattern” appearance in the macula, peripheral vascular leakage and ischemia. Sub-Tenon’s triamcinolone acetonide injection was administered in the superior temporal quadrant of the right eye. After two weeks, there was a decrease in CME, anterior chamber reaction and vitritis, therefore topical steroid was tapered.

**Figure 3:**
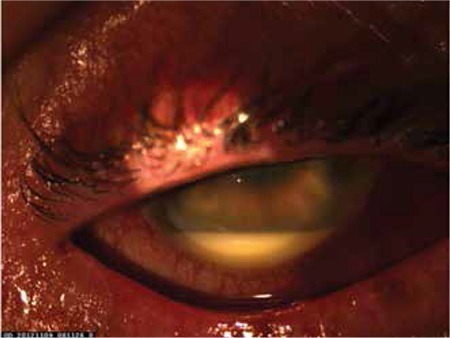
Severe anterior chamber reaction and hypopyon in the right eye

**Figure 4:**
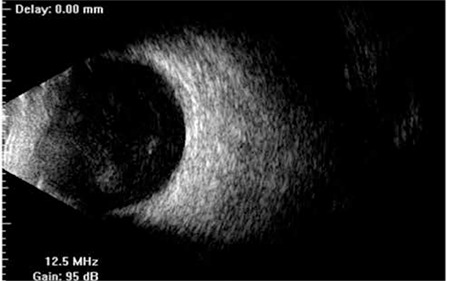
B-scan ultrasonography showing clear vitreous with no choroidal thickening and no exudative retinal detachment in the right eye

**Figure 5:**
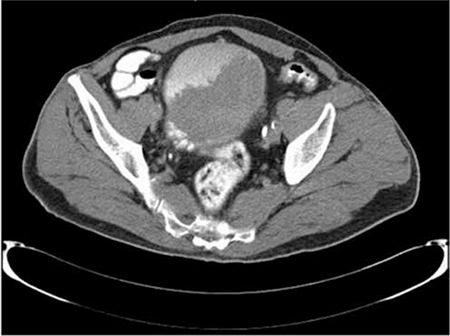
Abdominal computed tomography showing mass in the bladder

**Figure 6:**
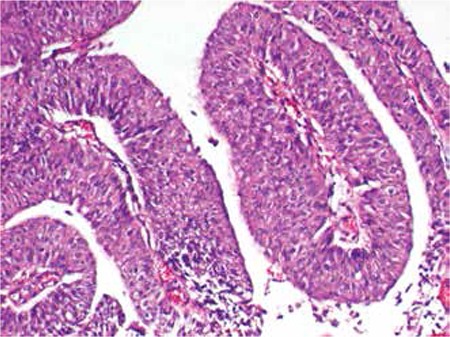
Hematoxylene and eosin staining of the bladder mass showing low grade papillary urethelial carcinoma with scarce atypical mitotic figures with no invasion. (x100)

The patient was lost to follow-up. Eight months later, the patient admitted to our clinic with vision loss in the right eye with severe anterior chamber reaction with fibrin stands and hypopyon (Figure 3). The right fundus could not be visualized due to hypopyon and fibrin strands in the anterior chamber. B-scan ultrasonography showed clear vitreous and no choroidal thickening (Figure 4). The patient was prescribed topical steroids, cycloplegics, subconjunctival steroid and antibiotics. He was diagnosed as recurrent chronic anterior uveitis. The oncology department was consulted for a systemic evaluation for a possible malignancy. Computed tomography of the abdomen revealed a mass in the bladder (Figure 5). Therefore, the patient was referred to the urology clinic. Biopsy of the mass confirmed bladder carcinoma. The patient underwent transurethral resection of the mass. Pathologic investigation revealed low grade papillary urethelial carcinoma (Figure 6). Intraocular inflammation decreased after the resection of the bladder mass and no recurrence of the uveitis attacks has been noted since. In his last examination, two years after the operation, the VA was light perception, seclusio pupilla was seen in biomicroscopic examination and clear vitreous was observed by ultrasonograpy.

## DISCUSSION

The incidence of true immune-mediated uveitis declines in the elderly. Infection endophthalmitis, especially arising after surgery, and malignancy occur at higher frequency. In this age group, the possibility of a masquerade or paraneoplastic syndrome should always be considered.^3,4^

We report a patient with bladder carcinoma whose initial symptoms led him to consult an ophthalmologist. The patient presented with hypopyon and anterior chamber reactions three times within a span of ten months.

Carcinomas predominate as the primary lesions that produce ocular metastases. Ocular metastases are hematogenously disseminated and most commonly affect the uveal tract. In our case, the presentation was in the form of a rather benign unilateral anterior uveitis without the involvement of the posterior segment, which misled us to a diagnosis of idiopathic anterior uveitis after an initial etiological investigation overlooking the possibility of a malignancy. The patient had no sign of an intraocular mass at initial presentation or during follow-up.

Initial clinical presentation of urinary bladder carcinoma with distant metastasis is rare.^4^ Urothelial metastases of the most common sites are regional lymph nodes, liver, lungs, and bone.^5^ To our knowledge, initial clinical presentation with ophthalmologic pathology such as anterior uveitis and hypopyon has not been previously reported for bladder carcinoma.

Intraocular inflammation presenting as hypopyon may occur in acute lymphoblastic leukemia (ALL), acute myeloid leukemia (AML), and chronic myeloid leukemia (CML). Isolated presentation of hypopyon uveitis is very rare in AML, with only two similar reported cases.^6^ Due to the patient’s age being over 60 years and isolated presentation of hypopyon and uveitis, the investigation of the patient for leukemia and lymphoma was recommended by other reports.^3,4^ There was no clinical or laboratory evidence of hematologic malignancy.

The term ‘masquerade syndrome’ refers to a wide variety of disorders whose clinical features include the presence of cells either in the anterior chamber, vitreous or both, but are unrelated to any immune-mediated uveitic diseases. Paraneoplastic retinopathies constitute a subgroup of this syndrome, which is defined as carcinoma-associated retinopathy (CAR) and melanoma-associated retinopathy. CAR patients usually present with bilateral vision loss due to extensive retinopathy and uveitis. Small-cell lung, gynecologic and breast cancers are diagnosed in most of the patients.^7^

The coexistence of bladder carcinoma and ocular inflammation in our patient may be explained by several possible pathophysiological mechanisms. The presence of bladder carcinoma with the anterior chamber reaction may be completely coincidental. Nevertheless, the cessation of ocular inflammatory findings after the resection of the tumor led us to search for a relationship between the two entities. The cessation of ocular inflammation despite the absence of a curative treatment for the ocular disease or a systemic chemotherapy supported the evidence of absence of any malignant cells in the ocular tissues and pointed to the remote effects of the malignancy presenting as ocular inflammation. The cytologic examination of the aqueous humor or the vitreous, which we failed to obtain, may be of great help in the diagnosis.

## CONCLUSION

To our knowledge, this is the first case of bladder carcinoma with an initial presentation of anterior uveitis. The case demonstrates the problems encountered in reaching a diagnosis and stresses the importance of a high index of suspicion of metastatic disease in elderly patients presenting with uveitis. Timely diagnosis is essential, particularly if the suspicion of ocular or systemic malignancy needs to be validated, as early diagnosis in ocular malignancy or metastasis can preserve sight and improve survival.

### Ethics

Informed Consent: It was taken.

Peer-review: Externally peer-reviewed.
